# Author Correction: Protein-peptide association kinetics beyond the seconds timescale from atomistic simulations

**DOI:** 10.1038/s41467-018-03452-0

**Published:** 2018-03-09

**Authors:** Fabian Paul, Christoph Wehmeyer, Esam T. Abualrous, Hao Wu, Michael D. Crabtree, Johannes Schöneberg, Jane Clarke, Christian Freund, Thomas R. Weikl, Frank Noé

**Affiliations:** 1 Department of Molecular and Cell Biology and California Institute for Quantitative Biosciences, University of California, Berkeley, CA 94720 USA; 2 Max Planck Institute of Colloids and Interfaces, Department of Theory and Bio-Systems, 14476 Potsdam, Germany; 3 Department of Chemistry, University of Cambridge, CB2 1EW Cambridge, UK; 4 Institute of Chemistry and Biochemistry, Freie Universität Berlin, Thielallee 63, 14195 Berlin, Germany

Correction to: *Nature Communications* 10.1038/s41467-017-01163-6, published online 23 October 2017

In the original version of this Article, the Acknowledgement section omitted financial support from the Deutsche Forschungsgemeinschaft grant SFB 958/A4. This error has now been corrected in both the PDF and HTML versions of the Article.

