# Supplementary material for: Protein-peptide association kinetics beyond the seconds timescale from atomistic simulations
Source: Nat Commun. 2017 Oct 23;8:1095. doi: 10.1038/s41467-017-01163-6 (PMC5653669; doi:10.1038/s41467-017-01163-6)
Supplement: Supplementary file 1 — Supplementary Information [file 41467_2017_1163_MOESM1_ESM.pdf]

Supplementary information

Protein-peptide association kinetics beyond the seconds timescale from atomistic simulations

Fabian Paul[1,3], Christoph Wehmeyer[1], Esam T. Abualrous[1], Hao Wu[1], Michael D. Crabtree[4], Johannes Schöneberg[1], Jane Clarke[4], Christian Freund[2], Thomas R. Weikl[3] and Frank Noé[1,*]

1: Department of Mathematics and Computer Science, Freie Universität Berlin, Arnimallee 6, 14195 Berlin, Germany
2: Department of Biochemistry, Freie Universität Berlin, Thielallee 63, 14195 Berlin, Germany
3: Max Planck Institute of Colloids and Interfaces, Department of Theory and Bio-Systems, 14476 Potsdam, Germany
4: Department of Chemistry, University of Cambridge, Cambridge, CB2 1EW, U.K.
*: Correspondence to frank.noe@fu-berlin.de

Suppl. Note 1 TRAMMBAR estimator

Suppl. Note 1.1 Optimization algorithm

The TRAMMBAR likelihood is minimized under the same constraints of detailed balance and probability normalization as the TRAM problem [1]. The optimal solution to the TRAMMBAR problem can be obtained by the fixed-point iteration of the following equations

$$v_i^{k,\mathrm{new}} := v_i^k \sum_j \frac{c_{ij}^k + c_{ji}^k}{\exp\left[f_j^k - f_i^k\right] v_j^k + v_i^k} \tag{1}$$

$$f_i^{k,\mathrm{new}} := -\ln \sum_{\mathbf{x}\in(X_{\mathrm{TRAM}}\cup X_{\mathrm{MBAR}})\cap S_i} \frac{\exp\left[-b^k(\mathbf{x})\right]}{\sum_l R_i^l \exp\left[f_i^l - b^l(\mathbf{x})\right]} \tag{2}$$

$$R_i^k := \sum_j \frac{\left(c_{ij}^k + c_{ji}^k\right) v_j^k}{v_j^k + \exp\left[f_i^k - f_j^k\right] v_i^k} + N_i^k - \sum_j c_{ji}^k + N_{\mathrm{MBAR}}^k \frac{e^{f^k}}{e^{f_i^k}} \tag{3}$$

where N_{MBAR}^k is the number of frames in X_{MBAR}^k. The equation for f_i^k from the TRAM estimator has to be extended to include a sum over X_{MBAR} as well as over X_{TRAM}: $e^{-f_i^k} = \sum_{\mathbf{x}\in(X_{\mathrm{TRAM}}\cup X_{\mathrm{MBAR}})\cap S_i} \exp\left[-b^k(\mathbf{x})\right] \mu(\mathbf{x})$ and we define $e^{-f^k} := Z^k = \sum_i e^{-f_i^k}$.

Like in TRAM, transitions in an isolated ensemble only need to be sampled in one direction as long as there is a second ensemble where the transition was sampled reversibly. The global equilibrium assumption that we make for the samples X_{MBAR} can be understood in the sense the dynamics that generated X_{MBAR} is infinitely fast and achieved global equilibrium. [2] This implies that all Markov states which were visited in X_{MBAR} are sampled reversibly. Hence, every transition that is irreversible in the unbiased MD simulation is implicitly made reversible if the end-states of that transition were visited in X_{MBAR}.

Suppl. Note 1.2 Relative influence of equilibrium data and time-correlated data

An important question in the application of TRAMMBAR is how much weight the datasets X_{TRAM} and X_{MBAR} each contribute to the estimate. Superficially, the answer to this question seems to be clear: for given simulation data, the weight is determined according to Eqs. (1-3) form the main text i. e. each

configuration x enters the likelihood with equal weight (or more precisely with a weight that is exactly given by $\mu(\mathbf{x})e^{f_i^k - b^k(\mathbf{x})}$ or $\mu(\mathbf{x})\, e^{f^k - b^k(\mathbf{x})}$ for $\mathbf{x} \in X_{\mathrm{TRAM}}$ or $\mathbf{x} \in X_{\mathrm{MBAR}}$ respectively). However, in the likelihood Eqs. (1-3) (main text) it is assumed that the configurations from X_{MBAR} have been drawn independently, and successive transitions in X_{TRAM} are independent. However in practice, neither assumption is fulfilled. [2] [2, 3] There is always some time correlation between the configurations generated by the replica exchange simulations and if the so-called "sliding window" method for counting transitions [4] is used, the transitions in X_{TRAM} are correlated. We solve this problem as follows: we assign an additional weight to each configuration. All configurations from X_{TRAM} are assigned the constant weight $g_{\mathrm{TRAM}}^{-1} := \Delta t_{\mathrm{TRAM}}/\tau$, where τ is the lag-time of the MEMM and Δt_{TRAM} is the saving interval of the configurations in X_{TRAM}. All configurations from X_{MBAR} are assigned the constant weight $1/\bar{g}_{\mathrm{MBAR}}$ where $g_{n,\mathrm{MBAR}}$ the is statistical inefficiency [5, 6] of the Markov state time series (discretized trajectory) of replica n and $\bar{g}_{\mathrm{MBAR}}$ is the average of $g_{n,\mathrm{MBAR}}$ over all replicas. The idea behind this is, that if lag-counting had been used and if the Markov property was fulfilled, successive transitions would be independent. Using the "sliding window" method, doesn't invalidate the Markov assumption (this is confirmed by the converging implied time scales and a successful Chapman-Kolmogorov test) but almost every transition, including its final configuration, is counted $g_{\mathrm{TRAM}} = \tau/\Delta t_{\mathrm{TRAM}}$ times more than it should. In X_{MBAR}, due to auto-correlation, every frame is counted about $\bar{g}_{\mathrm{MBAR}}$ times too much when using all the simulation data. So we include reweighting factors that compensate for this over-counting.

Equation (1) is not affected by the rescaling because c_{ij}^k scales like v_i^k such that the reweighting factor cancels. In equation (3), the substitutions $c_{ij}^k \to c_{ij}^k/g_{\mathrm{TRAM}}$, $v_i^k \to v_i^k/g_{\mathrm{TRAM}}$, $N_i^k \to v_i^k/g_{\mathrm{TRAM}}$, $N_{\mathrm{MBAR}}^k \to N_{\mathrm{MBAR}}^k/\bar{g}_{\mathrm{MBAR}}$ are made. In equation (2), the sum over $X_{\mathrm{TRAM}} \cup X_{\mathrm{MBAR}}$ is decomposed into two partial sums, one over X_{TRAM} and one over X_{MBAR}. Each partial sum is multiplied by its scaling factor.

Because the TRAMMBAR equations (as well as the TRAM equations) are invariant under a global multiplication of the data (i. e. if each configuration is counted n times), both scaling factors can be compiled into one factor $\bar{g}_{\mathrm{MBAR}}/g_{\mathrm{TRAM}}$, which only multiplies the values derived from X_{TRAM}.

Suppl. Note 2 Model system

We consider a two dimensional, x/y-periodic potential energy surface $E(x, y)$ with $x, y \in [-5, 5)$ (Suppl. Fig. 1 left), and a biased potential

$$B(x, y) = \max\{E(x, y),\, -0.5\, kT\} \tag{4}$$

where the potential wells are flattened (Suppl. Fig. 1 right). The dynamics are defined by a Metropolis Monte Carlo process which proposes moves ± 0.3 length units with uniform probability in each dimension. A set of 264 cluster centers is used to discretize the space into microstates and to define five macrostates representing the bound, intermediate, unbound, and two unspecifically bound states.

We compute a reference value for the binding free-energy $\Delta G^* = 5.64$ kT using

$$\Delta G = -\ln\left(\frac{\pi_{\mathrm{unbound}}}{1 - \pi_{\mathrm{unbound}}}\right) \tag{5}$$

Here, π_{unbound} is the sum of probabilities of states in the unbound set, and the probability weight of each microstate is approximated by $\exp(-E(x, y))$. To obtain a reference value for the mean first passage time (MFPT) of the dissociation, we spawn a series of 13780 simulations from within the bound state and run until a microstate of the unbound set is reached. The dissociation MFPT, $\mathrm{MFPT}^*_{\mathrm{off}} = 1820000 \pm 15000$ steps, is then computed as the arithmetic mean over the observed waiting times (Suppl. Fig. 2).

To compare the performance of TRAMMBAR to that of maximum likelihood MSMs, we perform a set of simulations for a fixed amount of data, build an MSM (only unbiased data) or an MEMM via TRAMMBAR (biased and unbiased data), and compute the dimensionless binding free-energy using equation (5) and the dissociation rate by

$$k_{\mathrm{off}} = \frac{1}{\mathrm{MFPT}\,(\mathrm{bound} \to \mathrm{unbound})} \tag{6}$$

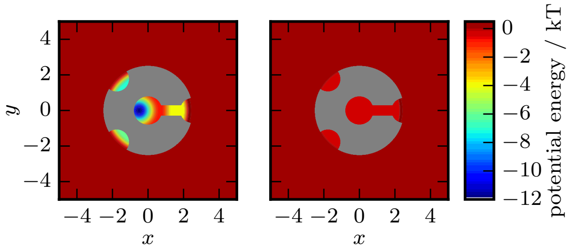

Suppl. Fig. 1: Unbiased (left) and biased (right) potential energy surfaces of the model system.

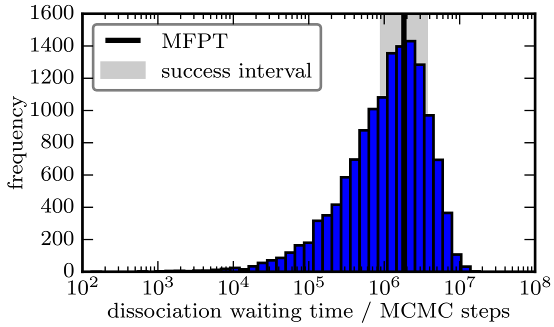

Suppl. Fig. 2: Distribution of dissociation waiting times from 13780 independent simulations from the bound state. The black line shows the mean value while the grey area denotes estimates which are considered successful in the main text.

where MFPT is the mean first passage time computed from the MSM of the unbiased ensemble.

As a successful estimate of the binding free-energy we define an estimate ΔG that is not farther than 1 kT away from the reference value ΔG^*, and a successful estimate of the dissociation MFPT is one that falls into the interval $[\frac{1}{2}\mathrm{MFPT}^*_{\mathrm{off}}, 2\mathrm{MFPT}^*_{\mathrm{off}}]$ where $\mathrm{MFPT}^*_{\mathrm{off}}$ is considered to be the exact time. To account for the intrinsic randomness of the kinetic Monte Carlo simulations, we repeat every set of simulations 100 times and compute the success probability for both estimators. For MSMs, we use multiple trajectories of $2 \cdot 10^6$ steps spawned in the bound state. For MEMMs, we use an equal amount of biased and unbiased data, where the unbiased trajectories are of length 10^3 steps and spawned in the unbound state, while the biased data consists of a single trajectory with a starting point drawn from the global equilibrium of the biased ensemble.

As the total amount of simulation data is increased, the success probability of both estimators converges to one (Fig. 4 in the main text). Estimates computed from the MEMM converge about two orders of magnitude

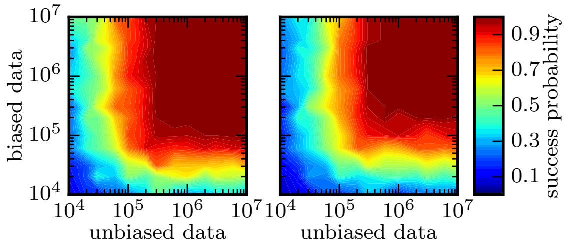

Suppl. Fig. 3: Success probability for ΔG (left) and $\mathrm{MFPT}_{\mathrm{off}}$ (right) as a function of the used amount of unbiased and biased data.

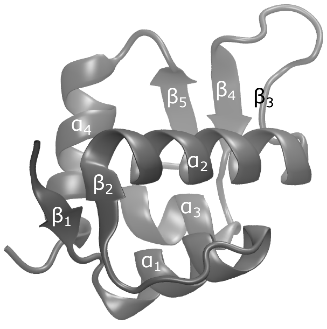

Suppl. Fig. 4: Crystal structure of the $^{25-109}$Mdm2 fragment with secondary structure labels (labels and Mdm2 atom coordinates reproduced from protein data bank file 3eqs [7]). Compared to the full-length N-terminal domain of Mdm2, the fragment lacks amino acids 1-24 that form a flexible lid. [8, 9, 10]

faster than estimates from the MSM; the performance of a direct sampling of dissociation times is roughly on par with the performance of MSMs, which is expected as the bottleneck for the estimation of the dissociation time is sampling the escape form the strongly bound state.

By varying the amounts of biased and unbiased data individually, we see that the MEMM estimates for ΔG and $\mathrm{MFPT}_{\mathrm{off}}$ are relatively insensitive to the data composition, as long as a sufficient amount of both data types is available (Suppl. Fig. 3). An interesting conclusion from this plot is that given a significant amount of biased data, an MEMM with a little bit of biased data added will perform much better than a plain MSM.

Suppl. Note 3 Mdm2-PMI simulations

Suppl. Note 3.1 Biased MD simulations

Accelerated molecular dynamics simulations [11] (aMD) were performed in a generalized ensemble. In each ensemble a particular energy bias was applied to the Lennard-Jones (LJ) $U = U_{LJ}^{\text{protein}-\text{ligand}}$ interaction of the protein and the peptide. The bias energy (boost potential) for ensemble k is

$$B^k(\mathbf{x}) = \begin{cases} 0 & U(\mathbf{x}) \geq E^k \\ \frac{\left(E^k - U(\mathbf{x})\right)^2}{\alpha^k + E^k - U(\mathbf{x})} & U(\mathbf{x}) < E^k \end{cases}$$

where $U(\mathbf{x})$ is the unbiased LJ interaction between protein and ligand as prescribed by the force field, $\mathbf{x} \in \mathbb{R}^{3N}$ is the molecular conformation and E^k, α^k are constants. The zeroth ensemble was left unbiased with the choice $E^0 = -\infty$, $\alpha^0 = 0$. For higher ensembles $1 \leq k < 14$, the constants were chosen by dividing the energy range of the LJ interaction $-250\,\mathrm{kJ\,mol^{-1}} \leq U \leq 0\,\mathrm{kJ}$ into 14 approximately equals intervals. The constants α^k were then chosen such that the minimum $\underline{U}^k$ of the biased LJ energy in ensemble k is the lower bound of interval number k. E^k for each ensemble were chosen to lie $20\,\mathrm{kJ\,mol^{-1}}$ above $\underline{U}^k$. Because the biased energy $U^k(U) = U + B^k(U)$ is a monotonic function of U, the minimum $\underline{U}^k$ of the biased energy is given by

$$\underline{U}^k = \underline{U} + \frac{(E^k - \underline{U})^2}{\alpha^k + E^k - \underline{U}}$$

where $\underline{U}$ is the minimal unbiased LJ interaction energy which was found to be $\underline{U} \approx -250\,\mathrm{kJ\,mol^{-1}}$ from direct MD simulations. This expression can be solved for $\alpha^k = \frac{(E^k - \underline{U})^2}{\underline{U}^k - \underline{U}} + \underline{U} - E^k$. Together with the above-given recipe for choosing $\underline{U}^k$ and E^k, this allows us to fix all parameters of the boost potentials. The reduced bias energy is given by $b^k(\mathbf{x}) = \beta B^k(\mathbf{x}) = \frac{B^k(\mathbf{x})}{R \cdot 300\,\mathrm{K}}$ where R is the ideal gas constant.

For the aMD simulations the period between successive calculations of long-range forces was set to one integration time step (4 fs). The aMD simulations were coupled using the Hamiltonian replica exchange (HREMD) protocol. [12] In the HREMD simulations, exchanges were only attempted between neighboring Hamiltonians every 0.5 ps. Initial conformations for the simulations were prepared as described in the Methods section of the main text but leaving the peptide in the binding pocket. Six independent HREMD runs were performed of lengths 1052, 2476, 1000, 1000, 1000 and 771 ns giving in total 7299 ns of aggregated simulation time per replica (102.186 μs summed over all replicas). Exchange acceptance probabilities were checked not to be below 0.2. Still the typical acceptance was above 0.35 for all but one run.

Suppl. Note 3.2 Mutation model

Experimental results of changes in the free-energy of binding upon mutation ($\Delta\Delta G$) are compared between experiment and simulation. Experimental values are given in reference [13]. The computations were done using first-order perturbation theory, following the ideas of [14, 15, 16]. The computational protocol used here was already successfully applied to compute relative binding free energies for the Barnase-Barstar complex. [17]

The unnormalized probabilities of the wild type to be in the associated or dissociated state are defined as

$$z^{\mathrm{wt}}_{\mathrm{associated}} = \sum_{\mathbf{x}\in S_A} \mu(\mathbf{x})$$

$$z^{\mathrm{wt}}_{\mathrm{dissociated}} = \sum_{\mathbf{x}\in S_D} \mu(\mathbf{x})$$

where S_A is the set of associated conformations here defined as all conformations where the minimal heavy-atom distance between PMI and Mdm2 is less or equal to 1.0 nm and S_D is the remainder of all dissociated conformations. $\mu(x)$ is the (conformation-wise) reweighting factor computed from MBAR and the sum is taken over all samples generated by the HREMD simulations.

For the mutant, the corresponding unnormalized probabilities are

$$z^{\mathrm{mut}}_{\mathrm{associated}} = \sum_{\mathbf{x}\in S_A} e^{-\beta U_{\mathrm{mut}}(\mathbf{x})+\beta U_{\mathrm{wt}}(\mathbf{x})}\mu(\mathbf{x})$$

$$z^{\mathrm{mut}}_{\mathrm{dissociated}} = \sum_{\mathbf{x}\in S_D} e^{-\beta U_{\mathrm{mut}}(\mathbf{x})+\beta U_{\mathrm{wt}}(\mathbf{x})}\mu(\mathbf{x}).$$

Here, $U_{\mathrm{wt}}(\mathbf{x})$ and $U_{\mathrm{mut}}(\mathbf{x})$ are the potential energies of the conformation $\mathbf{x}$ computed with implicit-solvent force field (details below). The change in binding free energy can then be computed to first order as

$$\Delta\Delta G = RT \ln \frac{z^{\mathrm{wt}}_{\mathrm{associated}}}{z^{\mathrm{wt}}_{\mathrm{dissociated}}} \frac{z^{\mathrm{mut}}_{\mathrm{dissociated}}}{z^{\mathrm{mut}}_{\mathrm{associated}}}$$

where $T = 300$ K and R is the ideal gas constant. The potential energies $U_{\mathrm{wt}}(\mathbf{x})$ and $U_{\mathrm{mut}}(\mathbf{x})$ were computed with the Amber software using the Amber-99SB-ILDN force field parameters [18] (that same that were used for the explicit solvent simulations) in combination with the generalized Born-Neck2 model [19] and the mbondi3 intrinsic radii [19]. This combination of force field and solvation model has been successfully applied to predict peptide folding preferences. [20] These computations are a pure reanalysis where the present trajectories are reprocessed with the "sander" module of the AMBER MD suite [21] to compute the potential energies. $U_{\mathrm{wt}}(\mathbf{x})$ was computed from the original trajectories after removing water and ion coordinates. For the mutant, trajectories were modified similarly as described in Refs. [22, 16]: for a given mutation the side chain of the relevant amino acid was cut back to the C_β-atom and the hydrogens of a methyl-group were added around the C_β-atom. The new value of the dihedral angle $C - C_\alpha - C_\beta - H_{\beta 1}$ was taken to be identical to the $C - C_\alpha - C_\beta - C_\gamma$ dihedral angle in the original conformation. All bond angles and bond lengths between C_β and the hydrogens of the methyl group were set to the equilibrium values of the Amber-99SB

force field. These replacements were done for all mutations and all frames of the HREMD trajectories (taking only one frame every nanosecond, which resulted in a total number of 97986 frames per mutation).

The method described here requires an implicit solvent force field to compute the mutant energies. Computing the energies with an explicit solvent force field would not be possible because the removal of the side chain leaves an energetically unfavorable void in the water. In the implicit solvent model the void is modeled automatically as a region of high dielectric constant that corresponds to water. Implicit solvent force fields have been shown to perform comparably to explicit solvent force fields in predicting binding free energies. [23]

To examine the importance of different PMI side chains for the different binding modes shown in main text Fig. 1, we compute the change in binding free energy upon mutation $\Delta\Delta G$ but with the probabilities $z^{\mathrm{mut}}_{\mathrm{associated}}$, $z^{\mathrm{wt}}_{\mathrm{associated}}$ of the associated state replaced by the probabilities z_i^{mut}, z_i^{wt} of PCCA state S_i. That is, we pretend that the only associated state of PMI and Mdm2 is the PCCA state S_i and ask how then the binding free energy would change if some PMI side chain was mutated to alanine. Results are shown for all combinations of macro-state and mutation in table 2.

Suppl. Note 3.3 MEMM weights and bootstrapping

We set $\bar{g}_{\mathrm{MBAR}}/g_{\mathrm{TRAM}} = 0.01$, which expresses that we trust the replica-exchange data in X_{MBAR} in estimating the thermodynamics. Note that that all direct information about the kinetics still come from transition counts made in the unbiased simulation data.

To compute the error bars for the transition probabilities in the Chapman-Kolmogorov test and for other observables, a bootstrap was performed. For each bootstrap sample one TRAMMBAR run is performed. The samples are generated by a hybrid bootstrap algorithm that combines a simple bootstrap with the stationary bootstrap of Politis *et al* [24]. From the unbiased simulation data whole trajectories are drawn with replacement. From the HREMD data, trajectory blocks of random length are drawn according to the stationary bootstrap algorithm. The block length parameter of the stationary bootstrap is chosen to be the mean statistical inefficiency [5] of the discretized trajectories in the HREMD data set (40 ns). The HREMD data are treated as multi-dimensional time series where each dimension corresponds to one replica. During bootstrap these dimensions are always kept together.

Suppl. Note 3.4 MEMM validation

The strongly connected set of Markov states can vary as the lag-time is changed because some transitions at the very beginning or the very end of the trajectories are not counted if the lag time is large. Also during bootstrap the connected set for a single sample can be smaller than the connected set that one would obtain when using all simulation data. However for the implied time scales test, results from different lag times must be compared and error estimates, that can currently be only estimated with the bootstrap technique, must be supplied. To resolve this problem, we extend every estimate of the transition matrix and its stationary distribution to the full state space and define $\pi_i = 0$ and $T_{ij} = T_{ji} = 0$ for every state i that is not in the connected set of microstates. To find the implied time scale we first compute the Rayleigh quotient

$$\tilde{\lambda}_i = \frac{\mathbf{r}_i^T \mathbf{\Pi} \mathbf{T} \mathbf{r}_i}{\mathbf{r}_i^T \mathbf{\Pi} \mathbf{r}_i}$$

where $\mathbf{r}_i$ is fixed and is the i'th right eigenvector of the MEMM transition matrix $\mathbf{T}$ computed at the reference lag-time of 150 ns and $\mathbf{\Pi}$ is a matrix with the extended stationary distribution on its diagonal. We then approximate the implied time scale with

$$t_i \approx -\frac{\tau}{\ln \tilde{\lambda}_i}$$

Plots of the implied time scales depending on the lag time are shown in Suppl. Fig. 5. The Chapman-Kolmogorov test of the unbiased ensemble of the MEMM estimated at 150 ns are shown in Suppl. Fig. 6.

PCCA state	PMI residues	Mdm2 residues
2	S2, Y6, W7, L10, P12	L54, G58, Y67, Q72, H96
3	Y6, S11, P12	E25, R97, Y104
5	F3, Y6, W7, N8, L10	L54, F55, G58, Y67, Q72, V93
6	F3, N8, L9	L54
8	S2, E5, W7	R65, Y67, Q72, H96
9	T1, S2, F3, Y6, L10, S11, P12	F55, G58, Q59, Y67, Q72, H96, R97, Y100
10	T1, S2, F3, E5, Y6, L9	L54, F55, Q72, H73, H96, R97
12	S2, F3, Y6, W7, N8, L9, L10, S11, P12	L54, I61, Q72, H96, Y100
13	S2, F3, Y6, W7, L10, S11, P12	L54, I61, Q72, H96
A	F3, Y6, W7, L10, S11, P12	L54, I61, Q72, H96
B	T1, S2, F3, Y6, W7, L10, S11, P12	K51, F55, Q72, V93, K94, H96
C	T1, F3, Y6, W7, L10, S11, P12	L54, Y67, Q72, H96
D	T1, F3, Y6, W7, L10, P12	L54, Y67, Q72, V93, H96, Y100
E	T1, S2, F3, E5, Y6, L9, S11, P12	L54, G58, Y67, Q72, H96, R97
F	T1, E5, Y6, N8, L9, S11, P12	L54, G58, Y67, D68, Q71, Q72, H73, I74, V93, K94, H96
G	F3, E5, Y6, W7, L9, S11, P12	Y67, Q72, H96, R97
H	T1, S2, F3, E5, Y6, W7, P12	L54, G58, Y67, Q72, H96
I	Y6, W7, L10, S11, P12	E25, T26, R97, Y100, T101, Y104

Suppl. Table 1: **Residues that form stable intermolecular contacts by PCCA state.** A stable intermolecular contact of molecule A is defined as a residue from molecule A that has a minimal heavy distance <0.35 nm with a probability of 0.5 or more to any other residue form molecule B.

PCCA	T1	S2	F3	E5	Y6	W7	N8	L9	L10	S11	P12
0	N.A	N.A	N.A	N.A	N.A	N.A	N.A	N.A	N.A	N.A	N.A
1	4.8	4.7	10.9	5.7	9.1	10.7	3.3	5.4	5.4	2.1	6.0
2	3.8	11.3	15.9	3.4	12.2	19.1	10.0	9.3	9.2	1.1	9.5
3	1.6	0.5	5.1	2.9	1.1	0.3	0.6	2.9	4.2	1.1	0.9
4	1.6	1.2	0.2	0.6	2.8	-0.0	3.9	3.6	4.2	1.5	1.9
5	6.0	0.5	10.1	5.2	8.9	9.7	0.2	4.8	5.8	1.8	1.8
6	-0.7	1.3	10.3	2.9	6.0	2.3	2.2	6.5	6.2	2.6	2.6
7	1.6	1.8	8.7	4.8	0.1	14.8	3.4	6.7	7.2	2.1	4.2
8	1.9	1.6	7.6	3.0	3.6	9.5	2.6	2.7	4.6	1.0	3.1
9	-0.3	0.9	8.9	-0.7	8.0	3.4	-0.5	4.5	4.1	-0.0	3.9
10	5.2	2.6	11.5	2.6	7.9	3.4	4.0	8.8	7.1	-0.1	3.6
11	2.2	-0.7	10.3	3.7	3.4	7.2	1.0	4.4	4.4	1.0	1.8
12	2.0	-0.3	9.7	1.1	4.5	7.4	0.7	1.2	1.4	-1.2	3.0
13	-0.7	0.2	5.4	0.7	-0.1	4.9	-0.0	0.2	3.3	-0.3	-0.5
A	-0.8	0.1	10.1	0.6	-0.2	7.7	-0.1	0.1	3.2	-0.2	-0.6
B	1.6	1.0	11.3	1.8	4.8	5.5	2.0	0.7	4.7	-0.9	3.7
C	0.2	0.3	5.9	1.6	5.6	3.7	-0.6	2.1	6.4	0.6	2.8
D	1.6	1.7	8.7	3.5	7.6	8.2	2.4	0.9	4.1	0.9	2.3
E	2.4	1.5	8.7	3.4	7.6	2.3	1.6	4.1	7.8	0.4	3.4
F	6.1	0.4	8.8	5.1	6.9	3.1	3.2	5.7	7.1	2.8	2.3
G	4.8	1.2	5.8	4.7	7.4	8.4	4.0	4.6	4.1	0.2	3.6
H	5.0	4.2	15.4	5.4	7.8	9.4	3.2	2.0	5.1	3.0	4.8
I	1.8	0.8	5.1	2.4	1.5	-0.3	-1.6	-0.9	-0.4	-0.3	1.3

Suppl. Table 2: **Stability changes of the PCCA states upon PMI mutation.** The table contains the values $RT\left[\ln(z^{\mathrm{wt}}_{S_i}/z^{\mathrm{wt}}_{\mathrm{dissociated}}) - \ln(z^{\mathrm{mut}}_{S_i}/z^{\mathrm{mut}}_{\mathrm{dissociated}})\right]$ in $\mathrm{kcal\,mol^{-1}}$ for all mutations and all PCCA sets S_i. $z^{\mathrm{mut}}_{S_i}$ is the (unnormalized) probability of PCCA set S_i for a PMI peptide where a given side chain was mutated to Alanine and $z^{\mathrm{wt}}_{S_i}$ the corresponding probability of the wild type. $z^{\mathrm{mut}}_{\mathrm{dissociated}}$ and $z^{\mathrm{wt}}_{\mathrm{dissociated}}$ are the probabilities of the dissociated state for mutant and wild type respectively. Estimates for PCCA state 0 were not computed, because the state was not visited in the biased simulations, so estimates would be very inaccurate.

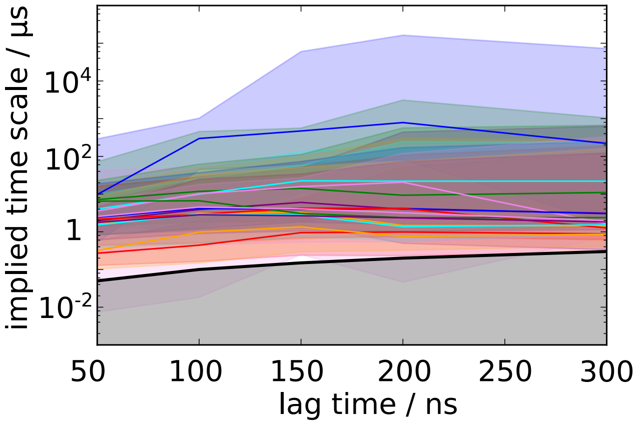

Suppl. Fig. 5: Implied time scales of the unbiased ensemble of the MEMM as a function of lag time. Transparently colored areas mark 95% confidence intervals.

To quantify how well the joint set of unbiased and biased simulations explored the conformational space, we check how the total number of macro-states seen in the simulation changes if the total amount of simulation data is varied. Results are shown in Suppl. Fig. 7. We make the conservative choice, that we only count a macro-state as being seen if the trajectory visits its core region. The core region of a macro-state is defined as the set of microstates that have PCCA memberships larger than 0.5. In our data, a core set defined in this way contains on average 58% of the microstates of the full PCCA set in which it is contained. Since there is no natural way to order the many short trajectories in our simulation data set and therefore no natural way to define the x first percent of the data, we pick a random order of the trajectories and repeat the analysis 100 times. Every realization is shown as a thin curve in Suppl. Fig. 7.

Suppl. Fig. 7 shows that the number of newly visited states has almost reached 100%, once 60% of the simulation data is included in the analysis. This indicates that the overwhelming majority of macro-states (that are metastable on timescales of the microsecond and longer) have been found in the simulation.

Suppl. Note 3.5 Coarse-graining and TPT analysis

The MEMM was coarse-grained into 15 macro-states. Macro-states 0 to 13 are defined with the PCCA+ algorithm [25] due to a small gap after the 13th implied time scales around $5\,\mu s$. The 15th macro-state is the set of all microstates that correspond to completely dissociated states (minimal heavy-atom distance between Mdm2 and PMI larger than 1.0 nm). The dissociated macro-state had to be defined manually, because it is of low metastability and is therefore not detected by the PCCA+ algorithm. (The life time of the dissociated state is only on the order of 10 ns.)

Transition path analysis [26, 27] was carried out for the reactive flux between the set of dissociated microstates and PCCA-state 13 which contains the crystal-like conformations.

The coarse-grained TPT flux between the 15 macro-states didn't contain enough information about the binding mechanism, because most of the TPT flux directly goes from the dissociated macro-state to PCCA-state 13 without any intermediates. Therefore we split PCCA-state 13 into 83 sub-states. The 83 sub-states were defined by applying the PCCA algorithm on the MSM of the unbiased ensemble and by taking the intersections between the new PCCA states with original PCCA state 13 as new (extra) macro-states. The resulting state definition is hierarchical: between the 14 top-level PCCA states, the kinetics happen on timescales of tens of microseconds or slower. Between the sub-states inside PCCA-state 13 the kinetics happen on the single-microsecond time scale or slower.

With the extended set of macro-states, the reactive flux between the set of the dissociated microstates and PCCA-state A (which contains the the crystal-like conformations) was computed with TPT. Results are shown in main text Fig. 3.

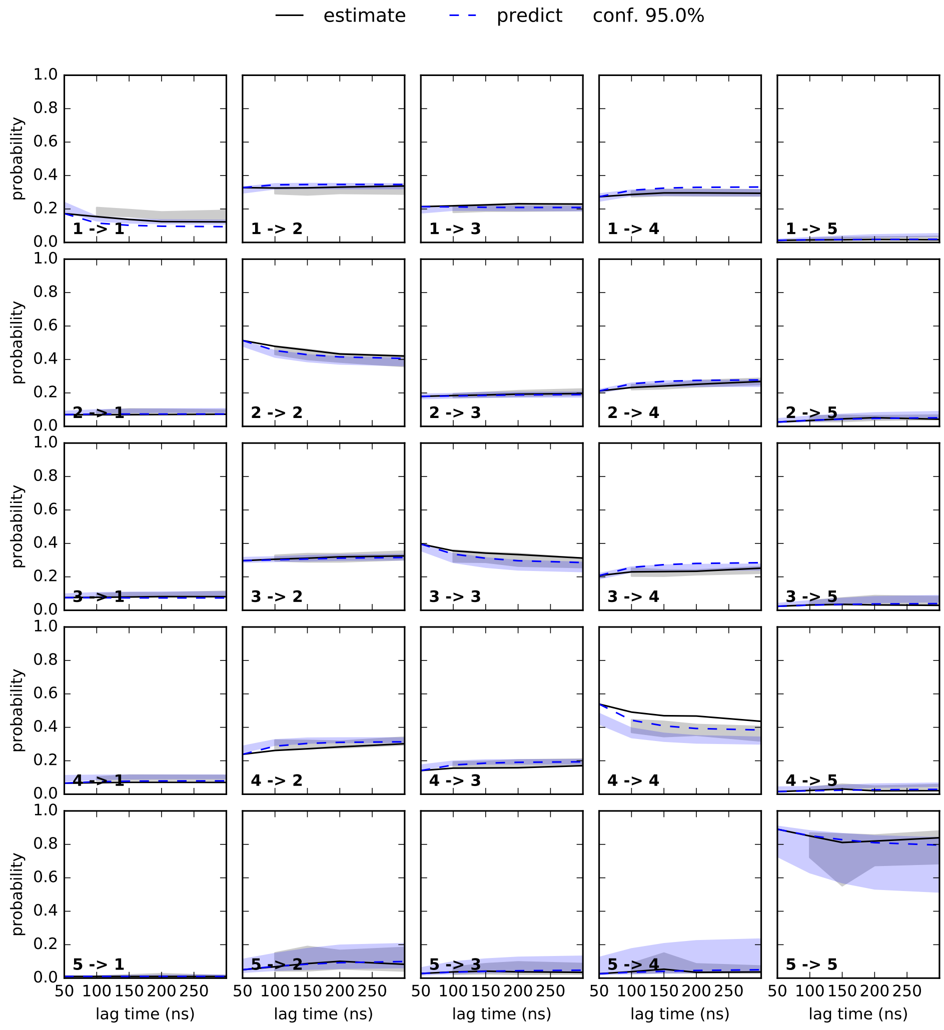

Suppl. Fig. 6: Chapman-Kolmogorov test of the unbiased ensemble of the full MEMM with 1056 microstates estimated at 150 ns using random test sets. Transparent regions represent 95% confidence intervals and were computed with bootstrap.

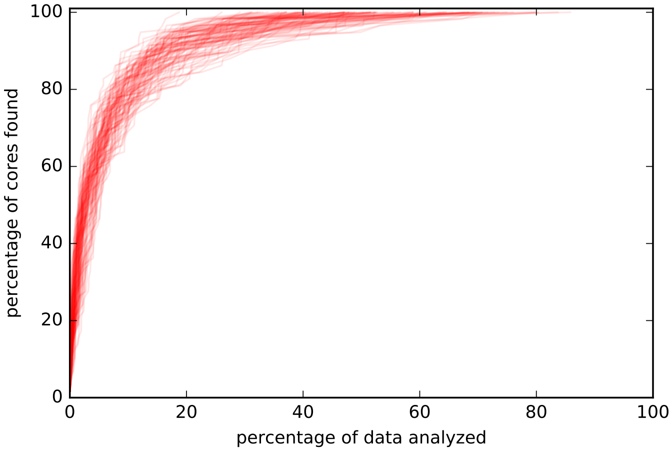

Suppl. Fig. 7: The faction of macro-states seen in the simulation depending on the total amount of simulation data analyzed. Every thin curve was computed from a different permutation of all simulation data (randomly mixing biased and unbiased data). The apparent convergence of the curves indicates that almost all metastable states have been discovered in the first 60% of the data. See Suppl. Note 3.4 for details.

In main text Fig. 1 we show only PCCA-states with a free energy that is not higher than $9\,\mathrm{kcal\,mol^{-1}}$ relative to that of macrostate A.

Suppl. Note 3.6 Computation of K_d and ΔG

We first define the associated state $S_{\mathrm{associated}}$ of the binding partners. For this definition we use the minimal heavy-atom distance d between PMI and Mdm2. Because of limited box size and periodic boundary conditions in the explicit-solvent MD, d can't exceed 3.5 nm. The histogram of d shows a minimum at 0.5 nm, is relatively constant between 1.0 nm and 1.8 nm and decreases to zero for larger values. Therefore we pick $d_{\max} = 1.0\,\mathrm{nm}$ as a conservative definition for the dissociated state and define the associated state as all points in phase space with $d \leq d_{\max}$. (The dependence of ΔG on the choice of $d_{\max}$ is shown in Suppl. Fig. 10a.) The volume $V_{\mathrm{associated}}$ of the associated state in position space is approximately $99.8\,\mathrm{nm}^3$. (Computed by counting water molecules that are beyond a distance of 1.0 nm from Mdm2 and subtracting their volume from the box volume.) The net solvent volume inside the simulation box is

$$V_{\mathrm{solvent}} = V_{\mathrm{box}} - V_{\mathrm{associated}} - V_{\mathrm{PMI}} = 352.43\,\mathrm{nm}^3$$

The effective concentration of non-interacting Mdm2 and PMI in the simulation box that can be used to translate simulation box quantities into molar quantities is then

$$C_{\mathrm{comput}} = [\mathrm{PMI}] = [\mathrm{MDM2}] = \frac{1}{N_A V_{\mathrm{solvent}}} = 4.71\,\mathrm{mM}$$

where N_A is Avogadro's constant. Then K_d can be computed by

$$K_d^{\mathrm{sim}} = \frac{p_{\mathrm{dissociated}}}{p_{\mathrm{associated}}} C_{\mathrm{comput}} = 0.34\,\mathrm{nM}$$

where $p_{\mathrm{associated}} = \sum_{x \in X_{(\mathrm{TRAM})\mathrm{MBAR}} \cap S_{\mathrm{associated}}} \mu(x)$, $p_{\mathrm{dissociated}} = 1 - p_{\mathrm{associated}}$ and $\mu(x)$ is the reweighting factor of simulation frame (conformation) x towards the unbiased ensemble computed with either MBAR or TRAMMBAR.

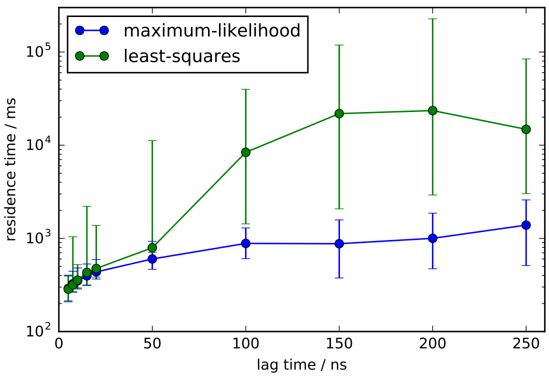

Suppl. Fig. 8: Lag time dependence of the residence time computed from the rate matrices that were estimated either according to the maximum-likelihood method from Refs.[28, 29] or the least-squared method from Ref.[30]. Error bars show 95% confidence intervals and were computed with bootstrap.

The standard free energy of binding is computed as

$$\Delta G = RT \ln \left(\frac{K_d^{\mathrm{sim}}}{1\,\mathrm{M}} \right)$$

where T is the temperature and R is the ideal gas constant.

Suppl. Note 3.7 Computational of association rate $k_{\mathrm{on}}^{\mathrm{MD}}$ and dissociation rate $k_{\mathrm{off}}^{\mathrm{MD}}$

To compute the association rate, we use the mean lifetime of the dissociated state (about 10 ns in the simulation box), and rescale it according to the volume of the dissociated state, resulting in an experimentally observable association rate of about $10^9\,\mathrm{M}^{-1}\mathrm{s}^{-1}$. For the computation of the residence time (or dissociation rate), ideally one would compute the mean first passage time (MFPT) from the strongly bound state to a state where the ligand is well separated from the protein. Because of the finite box size we have to choose a distance cutoff of $d_{\max} = 1.0\,\mathrm{nm}$ (see Suppl. Note 3.6). Therefore the ligand will have a certain probability p_{rebind} to rebind to the protein surface after reaching a separation of $d_{\max}$. We estimate this probability by running 10000 Brownian dynamics simulations of a point-like ligand, and counting how many time a ligand started at $d_{\max} + r_{\mathrm{PMI}} + r_{\mathrm{Mdm2}}$ diffuses to infinity and how many times it hits a sphere located at the origin with radius $r_{\mathrm{PMI}} + r_{\mathrm{Mdm2}}$. For $r_{\mathrm{PMI}} + r_{\mathrm{Mdm2}} = 0.63\,\mathrm{nm} + 1.08\,\mathrm{nm}$ (molecule radii computed from volumes $r = \sqrt[3]{4V/(3\pi)}$) we find $p_{\mathrm{rebind}} = 0.53$. This allows us to correct the residence time for the finite cutoff. We model the true dissociation to infinity as follows: every time the ligand reaches $d_{\max}$ it rebinds with a probability p_{rebind} and fully dissociates with a probability of $p_{\mathrm{escape}} = 1 - p_{\mathrm{rebind}}$. The (properly normalized) probability of escaping after n rounds of rebinding is $P_n = p_{\mathrm{rebind}}^n (1 - p_{\mathrm{rebind}})$. The expected dissociation time is therefore $T_{\mathrm{off}} = \sum_{n=0}^{\infty} T_{\mathrm{off}}^{\mathrm{MD}} (1+n) P_n = T_{\mathrm{off}}^{\mathrm{MD}} / p_{\mathrm{escape}}$ where $T_{\mathrm{off}}^{\mathrm{MD}}$ is the MFPT from the crystal-like bound state to the set of microstates with $d > d_{\max}$ measured from the MEMM (see below). In this calculation it is assumed that the binding partners are highly committed to form the crystal-like complex as soon as their surfaces meet and that binding in the simulation box is much faster than unbinding.

The residence time $T_{\mathrm{off}}^{\mathrm{MD}}$ is computed from the MEMM as the mean first passage time [26, 27] from the crystal-like bound state to the dissociated state with a separation $d > d_{\max}$ between PMI and MDM2. Computation of the MFPT $T_{\mathrm{off}}^{\mathrm{MD}}$ to the dissociated state is complicated by the fact that the dissociated state is not metastable on timescales comparable to the lag time of the MEMM, because of the small box size. At large lag times, the trajectory skips over the dissociated state, leading to an overestimation of MFPTs to the

dissociated state . [31] We addressed this problem by estimating rate matrices according to the maximum-likelihood method described in references [28, 29] and according to the least-squares method from reference [30] and subsequently computing the MFPT from these rate matrices. Rate matrices describe the dynamics in the limit of small lag times. Therefore estimating a rate matrix is a systematic way to extrapolate to a smaller lag time from data sampled at a larger lag time. In both rate matrix estimation methods we additionally imposed detailed balance of the rate matrix with respect to the TRAMMBAR equilibrium distribution of the unbiased ensemble. Furthermore for all pairs of states where no direct transition in either direction was observed in the unbiased MD trajectories, we constrained the corresponding forward and backward transition rates to zero. Both methods appear to converge at lag times of 150 ns, but the maximum-likelihood method [28, 29] gave the least dependence of the MFPT on lag time (Suppl. Fig. 8), so we propose this as a reference for $T_{\mathrm{off}}^{\mathrm{MD}}$. For comparison we redid the analysis described in Fig. 2b,d of the main text with the least-squares method [30]. Results are shown in Suppl. Fig. 9. We see that the least-squares method shows a higher variance than the maximum-likelihood method and gives higher values for the dissociation time. The higher values for the dissociation time are similar to those estimated from the transition matrix, however the transition-matrix MFPTs does not converge as a function of lag time.

The dependence of residence time on the choice of the cutoff used to define the dissociated state (d_{max}) is shown in Suppl. Fig. 10b.

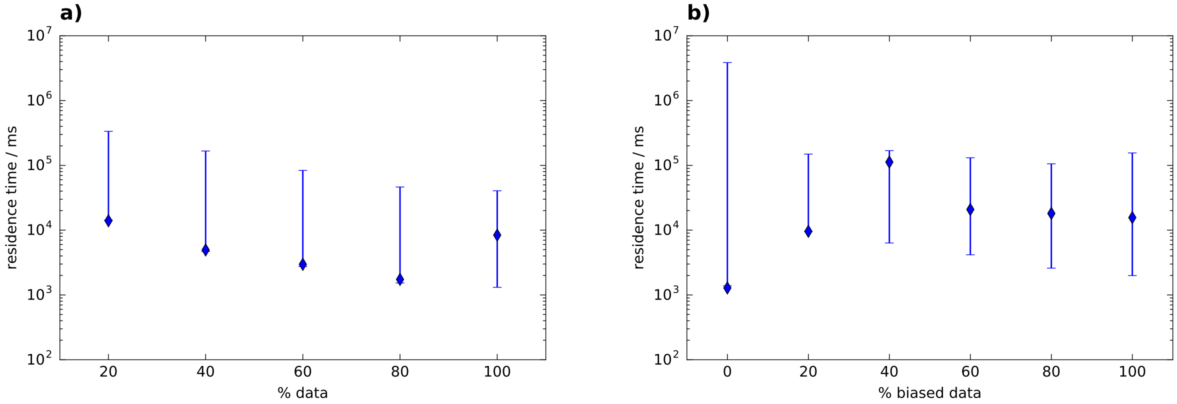

Suppl. Fig. 9: Same as the main text Fig. 2b,d but with rate matrices computed according to the least-squares method from [30]. **a:** Residence time as a function of the fraction of total data used in the estimation. **b:** Residence time as a function of the fraction of biased data used, while keeping the total amount of data constant. Diamonds mark the least-squares estimates, error bars indicate 95% confidence intervals that were computed with bootstrap.

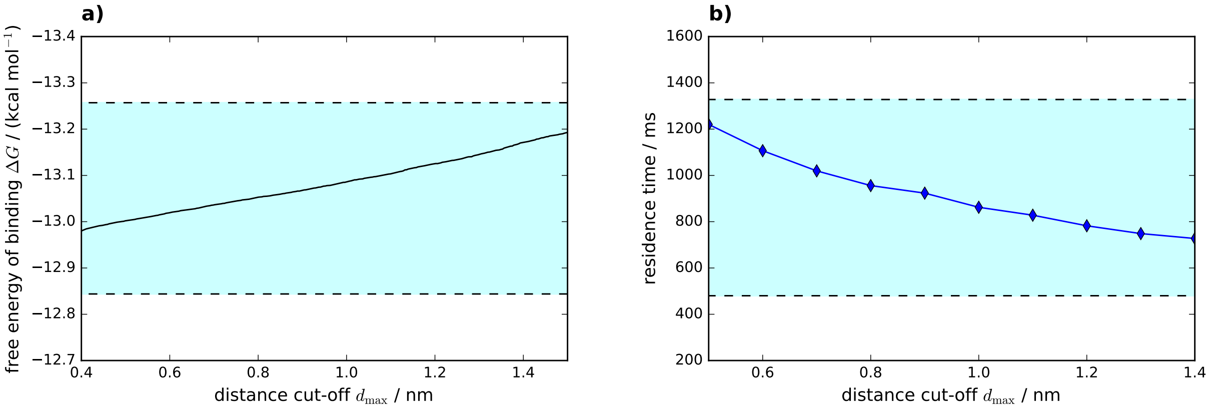

Suppl. Fig. 10: Dependence of simulation results for (a) the free energy of binding ΔG and (b) the complex residence time T_{off} from the cutoff value $d_{\max}$ that is used to define the dissociated state. All values are computed with correction for the volume of the dissociated state that corresponds to a given choice of $d_{\max}$ (as described in Suppl. Note 3.6 and Suppl. Note 3.7). The residence time is computed with the maximum-likelihood method from references [28, 29]. All variations are within the range of the statistical errors reported in the main text (cyan area bounded by the dashed lines).

Suppl. Note 4   Fluorescence anisotropy experiments

Suppl. Note 4.1   Protein expression, purification and refolding

The N-terminal domain of the Mdm2 protein (residues 25–109) was cloned into the pET21a vector and expressed in the *Escherichia coli* BL21(DE3). Cells were grown at 37 °C and induced with 1mM IPTG at an $OD_{600\,nm}$ of 0.8. After induction, the cells were cultured for an additional 12 hours at 37 °C before harvesting and the recombinant protein was purified from inclusion bodies as previously described. [32] The inclusion bodies were washed in PBS containing 0.05% Triton X-100, then solubilized in 6 M GnHCl in 100 mM Tris-HCl (pH 8.0), 1 mM EDTA, and 10 mM β-mercaptoethanol. The solubilized protein was dialyzed against 4 M GnHCl (pH 3.5) in 100 mM Tris, 1 mM EDTA, supplemented with 10 mM β-mercaptoethanol. Protein refolding was performed for 18 hours at 4 °C in 10 mM Tris-HCl (pH 7.0), 1 mM EDTA, and 10 mM β-mercaptoethanol. The protein was diluted (1:100) into the refolding buffer by adding in several sequential steps. To purify the protein, ammonium sulphate was added to a final concentration of 1.5 M and after 2 hours the mixture was centrifuged for 20 minutes at 10,000 rpm. The supernatant was then mixed with 10 ml of the Butyl Sepharose 4 Fast Flow. The protein was eluted with 100 mM Tris-HCl (pH 7.2) and 5 mM β-mercaptoethanol. The eluted protein was purified in a final step by gel filtration on HiLoad 26/60 Superdex 75 pg column with the running buffer containing 5 mM Tris-HCl (pH 8.0), 50 mM NaCl, and 10 mM β-mercaptoethanol.

Suppl. Note 4.2   Measurements of binding affinity and dissociation rate

Fluorescence anisotropy experiments were performed on a Multilabel 384-well plate reader (Tecan, Infinite M1000 PRO). The binding affinity of the PMI peptide towards the Mdm2 protein was determined using N-terminally fluorescently labelled peptide (fluorescein isothiocyanate, FITC-PMI), dissolved in a buffer containing 50 mM NaCl, 10 mM Tris pH 8.0, 1 mM EDTA, 10% DMSO. Varying concentrations (0-100 nM) of the empty Mdm2 protein were added to 10 nM (final concentration) of FITC-PMI in a final volume of 40 μl. The complex was then incubated for 15 minutes at room temperature and the binding was measured by fluorescence anisotropy. Data were fitted, and the K_d value was calculated using GraphPad Prism (Suppl. Fig. 11).

In order to compute the dissociation kinetics, binding competition experiments were conducted, starting with a mixture of 10 nM FITC-PMI peptide and 10 nM Mdm2 protein. Two such mixtures were prepared and incubated for 15 min at room temperature. Then 10 μM of unlabeled PMI peptide was added as competitor to one of the mixtures, while the other one was left untreated as a control, and the fluorescence anisotropy was simultaneously measured over time for both samples (main text Fig. 2f). The mixture with added PMI shows a decay due to unbinding of the FITC-PMI while the control shows no decay.

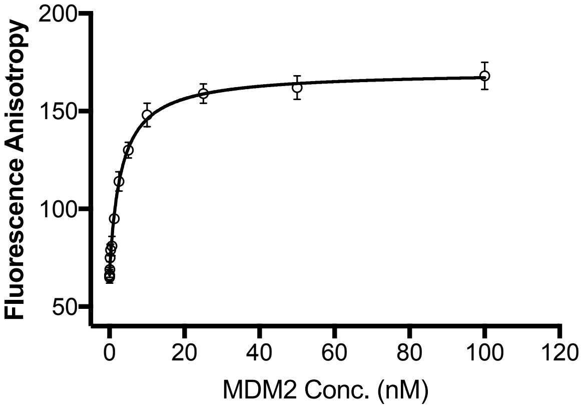

Suppl. Fig. 11: The binding curve of the empty Mdm2 protein titrated on FITC-PMI peptide.

To determine the measured dissociation rate and uncertainties, we fitted the time trace with a single-exponential model to find

$$k_{\text{off}}^{\text{exp}} = 0.037\,[0.030,\, 0.040]\ \text{s}^{-1},$$

The corresponding estimate for the residence time of the complex is

$$T_{\text{off}}^{\text{exp}} = 26.7\,[24.7,\, 34.1]\ \text{s}.$$

Suppl. Note 4.3 Stopped-flow experiments (association kinetics)

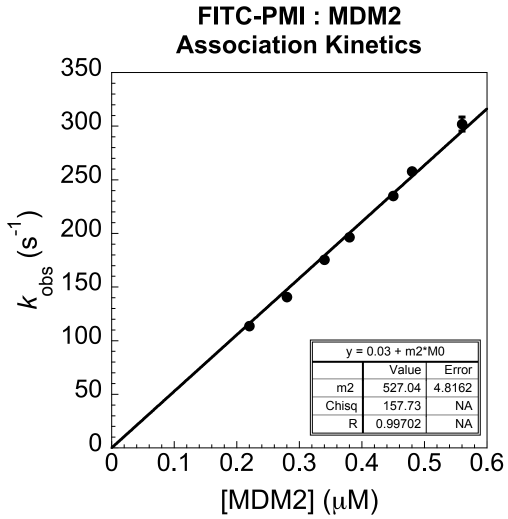

Suppl. Fig. 12: Observed rate constants (k_{obs}) from pseudo-first-order association binding experiments. The association rate constant (k_{on}) of $(5.27 \pm 0.05) \cdot 10^{8}\ \text{M}^{-1}\text{s}^{-1}$ was determined from the gradient of the straight-line fit. The intercept represents the dissociation rate constant (k_{off}), and was fixed to a value of $0.03\,\text{s}^{-1}$, as obtained in dissociation kinetics experiments. Error bars represent the standard error of the mean.

Biophysical buffer was 5 mM Tris, 50 mM NaCl, pH 8.0 with 10 mM 2-mercaptoethanol and 0.05 % Tween 20. FITC-PMI and Mdm2 were rapidly mixed using an SX20 stopped-flow spectrometer (Applied Photophysics). The temperature was maintained at 25°C, and an excitation wavelength of 493 nm, in conjunction with a 515 nm long-pass filter was utilized. Association experiments were performed under pseudo-first-order conditions, such that the concentration of Mdm2 (220 nM to 560 nM) was at least 10-fold higher than the concentration of FITC-PMI (20 nM). For each concentration of Mdm2, 10–19 traces were collected, and data within the dead time of mixing (the first 2 ms) were removed before analysis. Individual traces were fit to a single-exponential decay function to extract observed association rate constants (k_{obs}), as described previously. [33] Concentration dependent k_{obs} were fit to a straight line to extract the association rate constant (k_{on}). The intercept of the line represents the dissociation rate constant (k_{off}), and was fixed to the k_{off} obtained in the dissociation kinetics experiment ($0.03\,\text{s}^{-1}$). To ensure accurate concentrations in experiments, protein stock concentrations were determined from amino acid analysis (Department of Biochemistry, University of Cambridge, UK) and diluted by weight.

Suppl. References

[1] Wu, H., Paul, F., Wehmeyer, C. & Noé, F. Multiensemble Markov models of molecular thermodynamics and kinetics. *Proc. Natl. Acad. Sci. USA* **113**, E3221–E3230 (2016).

[2] Shirts, M. R. & Chodera, J. D. Statistically optimal analysis of samples from multiple equilibrium states. *J. Chem. Phys.* **129**, 124105 (2008).

[3] Trendelkamp-Schroer, B., Wu, H., Paul, F. & Noé, F. Estimation and uncertainty of reversible Markov models. *J. Chem. Phys.* **143**, 174101 (2015).

[4] Prinz, J.-H. *et al.* Markov models of molecular kinetics: Generation and validation. *J. Chem. Phys.* **134**, 174105 (2011).

[5] J. Grotendorst, A. M., D. Marx (ed.). *Quantum simulations of complex many-body systems: From theory to algorithms*, chap. Statistical analysis of simulations: data correlations and error estimation, 423–445 (NIC Series, 2002).

[6] Chodera, J. D., Swope, W. C., Pitera, J. W., Seok, C. & Dill, K. A. Use of the weighted histogram analysis method for the analysis of simulated and parallel tempering simulations. *J. Chem. Theory Comput.* **3**, 26–41 (2007).

[7] Pazgier, M. *et al.* Structural basis for high-affinity peptide inhibition of p53 interactions with MDM2 and MDMX. *Proc. Natl. Acad. Sci. USA* **106**, 4665–4670 (2009).

[8] Showalter, S. A., Bruschweiler-Li, L., Johnson, E., Zhang, F. & Brüschweiler, R. Quantitative lid dynamics of MDM2 reveals differential ligand binding modes of the p53-binding cleft. *J. Am. Chem. Soc.* **130**, 6472–6478 (2008).

[9] Bueren-Calabuig, J. A. & Michel, J. Elucidation of ligand-dependent modulation of disorder-order transitions in the oncoprotein MDM2. *PLoS Comput. Biol.* **11**, 1–27 (2015).

[10] Bueren-Calabuig, J. A. & Michel, J. Impact of Ser17 phosphorylation on the conformational dynamics of the oncoprotein MDM2. *Biochemistry-US* **55**, 2500–2509 (2016).

[11] Hamelberg, D., Mongan, J. & McCammon, J. A. Accelerated molecular dynamics: A promising and efficient simulation method for biomolecules. *J. Chem. Phys.* **120**, 11919–11929 (2004).

[12] Fukunishi, H., Watanabe, O. & Takada, S. On the Hamiltonian replica exchange method for efficient sampling of biomolecular systems: Application to protein structure prediction. *J. Chem. Phys.* **116**, 9058–9067 (2002).

[13] Li, C. *et al.* Systematic mutational analysis of peptide inhibition of the p53-MDM2/MDMX interactions. *J. Mol. Biol.* **398**, 200–213 (2010).

[14] Matysiak, S. & Clementi, C. Optimal combination of theory and experiment for the characterization of the protein folding landscape of S6: How far can a minimalist model go? *J. Mol. Biol.* **343**, 235–248 (2004).

[15] Matysiak, S. & Clementi, C. Minimalist protein model as a diagnostic tool for misfolding and aggregation. *J. Mol. Biol.* **363**, 297–308 (2006).

[16] Zeller, F. & Zacharias, M. Efficient calculation of relative binding free energies by umbrella sampling perturbation. *J. Comput. Chem.* **35**, 2256–2262 (2014).

[17] Plattner, N., Doerr, S., De Fabritiis, G. & Noé, F. Complete protein-protein association kinetics in atomic detail revealed by molecular dynamics simulations and Markov modelling. *Nat. Chem.* (2017). Advance online publication, DOI: 10.1038/nchem.2785.

[18] Lindorff-Larsen, K. *et al.* Improved side-chain torsion potentials for the Amber ff99SB protein force field. *Proteins* **78**, 1950–1958 (2010).

[19] Nguyen, H., Roe, D. R. & Simmerling, C. Improved generalized Born solvent model parameters for protein simulations. *J. Chem. Theory Comput.* **9**, 2020–2034 (2013).

[20] Maffucci, I. & Contini, A. An updated test of AMBER force fields and implicit solvent models in predicting the secondary structure of helical, β-hairpin, and intrinsically disordered peptides. *J. Chem. Theory Comput.* **12**, 714–727 (2016).

[21] Case, D. *et al.* AMBER 2015. Tech. Rep., University of California, San Francisco (2015).

[22] Massova, I. & Kollman, P. A. Computational alanine scanning to probe protein-protein interactions: A novel approach to evaluate binding free energies. *J. Am. Chem. Soc.* **121**, 8133–8143 (1999).

[23] Zeller, F. & Zacharias, M. Evaluation of generalized Born model accuracy for absolute binding free energy calculations. *J. Phys. Chem. B* **118**, 7467–7474 (2014).

[24] Politis, D. N. & Romano, J. P. The stationary bootstrap. *J. Am. Stat. Assoc.* **89**, 1303–1313 (1994).

[25] Röblitz, S. & Weber, M. Fuzzy spectral clustering by PCCA+: application to Markov state models and data classification. *Adv. Data Anal. Classif.* **7**, 147–179 (2013).

[26] Noé, F., Schütte, C., Vanden-Eijnden, E., Reich, L. & Weikl, T. R. Constructing the equilibrium ensemble of folding pathways from short off-equilibrium simulations. *Proc. Natl. Acad. Sci. USA* **106**, 19011–19016 (2009).

[27] Metzner, P., Schütte, C. & Vanden-Eijnden, E. Transition path theory for Markov jump processes. *Mult. Mod. Sim.* **7**, 1192–1219 (2009).

[28] Kalbfleisch, J. D. & Lawless, J. F. The analysis of panel data under a Markov assumption. *J. Am. Stat. Assoc.* **80**, 863–871 (1985).

[29] McGibbon, R. T. & Pande, V. S. Efficient maximum likelihood parameterization of continuous-time Markov processes. *J. Chem. Phys.* **143**, 034109 (2015).

[30] Crommelin, D. & Vanden-Eijnden, E. Data-based inference of generators for Markov jump processes using convex optimization. *Multiscale Model. Sim.* **7**, 1751–1778 (2009).

[31] Suárez, E., Adelman, J. L. & Zuckerman, D. M. Accurate estimation of protein folding and unfolding times: Beyond Markov state models. *J. Chem. Theory Comput.* **12**, 3473–3481 (2016).

[32] Czarna, A. *et al.* High affinity interaction of the p53 peptide-analogue with human Mdm2 and Mdmx. *Cell Cycle* **8**, 1176–1184 (2009).

[33] Crabtree, M. D. *et al.* Conserved helix-flanking prolines modulate intrinsically disordered protein:target affinity by altering the lifetime of the bound complex. *Biochemistry-US* **56**, 2379–2384 (2017).
